# Transcriptional Regulation of Stearoyl-Acyl Carrier Protein Desaturase Genes in Response to Abiotic Stresses Leads to Changes in the Unsaturated Fatty Acids Composition of Olive Mesocarp

**DOI:** 10.3389/fpls.2019.00251

**Published:** 2019-03-05

**Authors:** M. Luisa Hernández, M. Dolores Sicardo, Miguel Alfonso, José M. Martínez-Rivas

**Affiliations:** ^1^Instituto de la Grasa (IG-CSIC), Seville, Spain; ^2^Estación Experimental de Aula Dei (EEAD-CSIC), Zaragoza, Spain

**Keywords:** *Olea europaea*, olive, abiotic stress, gene expression, unsaturated fatty acids, stearoyl-ACP desaturase

## Abstract

In higher plants, the stearoyl-acyl carrier protein desaturase (SAD) catalyzes the first desaturation step leading to oleic acid, which can be further desaturated to linoleic and α-linolenic acids. Therefore, SAD plays an essential role in determining the overall content of unsaturated fatty acids (UFA). We have investigated how *SAD* genes expression and UFA composition are regulated in olive (*Olea europaea*) mesocarp tissue from Picual and Arbequina cultivars in response to different abiotic stresses. The results showed that olive *SAD* genes are transcriptionally regulated by temperature, darkness and wounding. The increase in *SAD* genes expression levels observed in Picual mesocarp exposed to low temperature brought about a modification in the UFA content of microsomal membrane lipids. In addition, darkness caused the down-regulation of *SAD* genes transcripts, together with a decrease in the UFA content of chloroplast lipids. The differential role of olive *SAD* genes in the wounding response was also demonstrated. These data point out that different environmental stresses can modify the UFA composition of olive mesocarp through the transcriptional regulation of *SAD* genes, affecting olive oil quality.

## Introduction

Unsaturated fatty acids not only serve as a major source of reserve energy in the form of triacylglycerols, but also constitute complex lipids that are essential components of cellular membranes. In plants, an increasing number of studies has also proposed UFA and their derivatives as signaling molecules, which are involved in the response to biotic and abiotic stresses (Kachroo and Kachroo, 2009).

In higher plants, *de novo* fatty acid biosynthesis starts in the plastid by successive addition of two carbon atoms from acetyl-CoA, mainly leading to the synthesis of palmitoyl-acyl carrier protein (palmitoyl-ACP) and stearoyl-ACP (Harwood, 2005). These products, which can be desaturated in different cellular compartments, are the source of most of the fatty acids present in plant lipids. The first desaturation takes place in the plastid by the action of the Δ9 stearoyl-ACP desaturase (SAD), which produces oleoyl-ACP, using ferredoxin as electron donor. The oleoyl-ACP is then cleaved by specific thioesterases to free fatty acids, which are then incorporated into glycerolipids, where can be further desaturated to linoleic and α-linolenic acid by membrane-bound fatty acid desaturases, that differ in their cellular localization, lipid substrates, and electron donor system (Shanklin and Cahoon, 1998). The microsomal oleate desaturase (FAD2) and linoleate desaturase (FAD3) are located in the endoplasmic reticulum, use phospholipids as acyl substrates and NADH, NADH-cytochrome b_5_ reductase and cytochrome b_5_ as electron donor system. On the other hand, the plastidial oleate desaturase (FAD6) and linoleate desaturase (FAD7/8) are located in the plastid, use glycolipids as acyl carriers and NAD(P)H, ferredoxin-NAD(P) reductase and ferredoxin as electron donor system (Supplementary Figure S1).

Plant fatty acid desaturases are regulated by different environmental and physical stresses. Temperature is one of the main environmental factors affecting fatty acid desaturases. The plants ability to adjust membrane lipid fluidity in response to temperature by changing the levels of UFA is allowed by the regulated activity of fatty acid desaturases (Iba, 2002; Upchurch, 2008). Several mechanisms have been described to explain how temperature regulates fatty acids desaturation, including transcriptional (Kargiotidou et al., 2008) and post-transcriptional regulation (Matsuda et al., 2005), and through the effect of temperature on oxygen availability (Rolletschek et al., 2007). Light is a second environmental factor regulating fatty acid desaturation. An increase in polyunsaturated fatty acids have been reported in cucumber cotyledons (Murphy and Stumpf, 1979), oat leaves (Ohnishi and Yamada, 1983), and Arabidopsis callus cultures (Brockman et al., 1990) exposed to light. Regarding the molecular mechanism by which light regulates fatty acid desaturase, transcriptional (Kargiotidou et al., 2008) and post-translational (Collados et al., 2006) regulation have been reported. Plant fatty acid desaturases are also affected by wounding or pathogen attack. In fact, plants have evolved multiple mechanism to defend themselves against pathogens. It has been widely described the key role of polyunsaturated fatty acids in plants defense response, mainly as precursor of signal molecules such as jasmonic acid (Farmer, 1994). Accordingly, the transient induction of fatty acid desaturases in response to wounding has been observed in several plants (Hamada et al., 1996; Nishiuchi et al., 1997).

Olive (*Olea europaea* L.) is one of the first plants to be cultivated for oil production, and olive oil is the one with most impact in the Mediterranean region either at the economic, social and cultural levels (Baldoni et al., 2009). Virgin olive oil is a natural fruit juice, highly enriched in oleic acid (55–83%), while linoleic acid accounts for 3.5–21%, and linolenic acid for less than 1%. The relative contents of these UFA depends mainly on the olive cultivar, but also on pedoclimatic and culture conditions (Beltrán et al., 2004), affecting the nutritional (Perona et al., 2005) and technological properties (Aparicio et al., 1999) of the oil, and, therefore, the olive oil quality. In olive, three genes encoding SAD have been reported (Haralampidis et al., 1998; Parvini et al., 2016). *SAD2* was highly expressed in mesocarp and seed, whereas the transcript of *SAD3* was mainly detected in young drupes and leaves. In contrast, *SAD1* expression levels remained low in all tissues studied. In addition, *SAD2* gene has been suggested as the main contributor to oleic acid synthesis in olive mesocarp (Parvini et al., 2016). Regarding membrane desaturases, two genes encoding microsomal (Hernández et al., 2005) and one plastidial (Banilas et al., 2005; Hernández et al., 2011) oleate desaturases have been described, identifying *FAD2-2* as the main gene responsible for the linoleic acid accumulation in olive mesocarp (Hernández et al., 2009). Finally, four genes encoding linoleate desaturases have been reported, two microsomal (Banilas et al., 2007; Hernández et al., 2016) and two plastidial (Poghosyan et al., 1999; Hernández et al., 2016), with *FAD7-1* and *FAD7-2* genes being suggested to contribute mostly to the linolenic acid present in the olive mesocarp (Hernández et al., 2016).

Early studies using olive callus cultures revealed that FAD2 is regulated by temperature and light intensity, while FAD7 is affected by high temperature (Hernández et al., 2008). More recently, transcriptional analysis conducted on olive mesocarp exposed to low and high temperatures demonstrated the role of *FAD2*, *FAD6*, and *FAD7* genes in regulating UFA levels in order to maintain the fluidity of the biological membranes (Hernández et al., 2011; Matteucci et al., 2011; D’Angeli et al., 2013). In the same way, RNAseq analysis has shown that *FAD2-2* gene increased its expression in olive leaves in response to cold (Leyva-Pérez et al., 2015). Furthermore, Hernández et al. (2011) observed a decrease in oleate desaturase genes expression levels when the olive fruit were incubated under darkness conditions. On the other hand, a transient induction of oleate desaturases, together with a slight increase in linoleic acid and the appearance of palmitolinoleic acid, have been reported in olive mesocarp subjected to wounding (Hernández et al., 2011) (Supplementary Figure S1). In addition, Padilla et al. (2012, 2014) described the transient induction of 13- and 9-lipoxygenases, and 13-hydroperoxide lyase in mechanically damaged olive mesocarp. These results showed the involvement of olive oleate desaturases in plant defense response providing the substrates to the different lipoxygenases pathways, including that of jasmonic acid synthesis (Weber, 2002). Not only that, a transcriptomic approach has also been performed to study the molecular interaction between the olive fruit fly (*Bactrocera oleae*) and tolerant and susceptible olive cultivars (Corrado et al., 2012; Grasso et al., 2017).

However, with the exception of a recent study on the effect of irrigation in desaturase gene expression of olive mesocarp (Hernández et al., 2018), there is still scarce information about the regulation of *SAD* genes in response to environmental stresses in plant tissues and, even more, in the case of the mesocarp of oil fruits. This tissue possesses the remarkable characteristic of having a high proportion of active chloroplasts together with a high amount of oil (Sánchez, 1994). In addition, SAD is of particular interest because is a key determinant of the overall level of fatty acid desaturation (Shanklin and Somerville, 1991), since this enzyme carries out the first desaturation step leading to oleic acid, which can be further desaturated to linoleic acid and α-linolenic acid. Therefore, it has a significant effect on the fluidity and rigidity of membrane system and the relationship of this to the adaption of plants to various environmental conditions.

For these reasons, we have studied in this work the transcriptional regulation of *SAD* genes in olive fruit remaining in branches incubated under different abiotic stresses, together with its impact at the metabolite level on the UFA content in different lipid classes of mesocarp tissue from Picual and Arbequina cultivars.

## Materials and Methods

### Plant Material and Stress Treatments

Olive (*Olea europaea* L. cv. Picual and Arbequina) trees were grown in the experimental orchard of Instituto de la Grasa, Seville (Spain), with drip irrigation and fertirrigation from the time of complete flowering to fruit ripening.

Four olive branches carrying 100 olive fruit at 28 WAF (turning stage) each one, were collected from different olive trees located in the same field, and transferred to growth chamber where they were incubated at 25°C with a 12 h light/12 h dark cycle, with a light intensity of 300 μmol m^−2^ s^−1^. These incubation parameters attempted to simulate physiological conditions of the tree, and were considered the standard conditions. No significant alterations in the fatty acid composition or *SAD* genes expression levels were observed in the mesocarp tissue when olive fruits were incubated under the above mentioned standard conditions (Supplementary Figure S2). For stress treatments, a new set of four olive branches with the same characteristics as previously mentioned were collected from the different olive trees and transferred to growth chamber, where the described standard conditions were altered to achieve the stress conditions to be studied. To examine the effect of temperature, the olive branches containing the fruits were incubated at 15°C for the low temperature experiment and 35°C for the high temperature one, at the same standard light intensity. For darkness treatment, light was turned off maintaining the same standard temperature. To study the effect of wounding, the whole surface of the olive fruit was mechanically damaged at zero time exerting pressure using tweezers with serrated tips, so that mesocarp tissue was affected. Each experiment corresponding to a different treatment was carried out at a different day of the same week, to ensure that the olive fruits of the different experiments were in the same stage (28 WAF). Zero time was designated 2 h after the beginning of the light period in every experiment, in order to maintain the natural photoperiod day/night of the olive fruit. At the indicated times, 10 olive fruits were taken from each olive branch, then 1–2 g of olive mesocarp was collected from 5 different olive fruits for RNA isolation, and 1.5 g was collected from the other 5 different olive fruits for lipid analysis. Therefore, in each experiment we used approximately 60 olive fruits from three olive branches, one branch for each biological replicate. In all experiments we incubated four branches, so that in case that we had a problem with a branch, to ensure we had another branch with olives and we could continue with the experiment. Olive mesocarp samples were frozen in liquid nitrogen and stored at −80°C.

### Total RNA Extraction and cDNA Synthesis

Total RNA isolation was carried out according to Hernández et al. (2005) using 1–2 g of frozen olive fruit mesocarp tissue collected from at least five different olive fruit per each of the three biological replicates. Briefly, the frozen olive mesocarp was ground in a pre-cooled mortar with liquid nitrogen and homogenized with the extraction buffer, containing Tris-HCl, NaCl, Na_2_EDTA and SDS, and 2-mercaptoethanol. Afterward, nucleic acids were extracted with phenol/chloroform twice, and precipitated with NaAc and ethanol. The nucleic acid pellet was resuspended in DEPC treated water and LiCl was added to precipitate the RNA. The pellet was washed twice with 70% ethanol and resuspended in 25 μl DEPC-water. RNA quality verification, removal of contaminating DNA and cDNA synthesis were performed as described by Hernández et al. (2009), using the TURBO DNA-free kit (Ambion, United States) and SuperScript III First-Strand Synthesis System (Invitrogen, Carlsbad, CA, United States) with oligo (dT)_20_.

### Quantitative Real Time-PCR (qRT-PCR)

Gene expression analysis was performed by quantitative real time PCR (qRT-PCR) using a CFX Connect real-time PCR System and iTaq Universal SYBR Green Supermix (Bio-Rad, Hercules, CA, United States). Primers for gene-specific amplification were described by Parvini et al. (2016) and are shown in Supplementary Table S1. Reaction mix (10 μL per well) contained 1X iTaq-QPCR Master Mix, 100 nM forward and reverse primers, and 2 μL of cDNA of appropriate dilution, which was selected according to the primers amplification efficiency. The thermal cycling conditions included an initial denaturation step of 95°C for 10 min, followed by 40 cycles of 95°C for 30 s, 60°C for 1 min, and 72°C for 30 s. The melting reaction from 55°C through 95°C, at 0.1°C s^−1^, following the final step of the PCR, was used to examine the specificity of the PCR amplification and the presence of primer dimers. Additionally, the purity of the PCR products were also checked by agarose gel electrophoresis. PCR efficiencies (*E*) of all primers were calculated using dilution curves with eight dilution points, twofold dilution, and the equation *E* = [10^(−1/slope)^] – 1. For normalization of the data, the olive ubiquitin2 gene (*OeUBQ2*, AF429430) was used as an endogenous reference. The qRT-PCR data were calibrated relative to the corresponding gene expression level at zero time for each treatment and cultivar, following the relative quantification by the 2^−ΔΔCt^ method (Livak and Schmittgen, 2001). The data are presented as means ± SD of the three biological replicates, each having two technical replicates per 96 well plate.

### Lipid Extraction and Fatty Acid Analysis

For lipid analysis, 1.5 g of frozen olive mesocarp tissue collected from at least five different olive fruits per each of the three biological replicate, were used. Olive fruit mesocarp tissue was firstly treated with isopropanol at 70°C for 30 min to inactivate endogenous lipase activity. Extraction of lipids was carried out according to Hara and Radin (1978), followed by their separation by thin layer chromatography as described by Hernández et al. (2008). Acid-catalyzed transmethylation of the different lipid preparations was performed to obtain the corresponding fatty acid methyl esters (Garcés and Mancha, 1993), which were analyzed by gas chromatography (Román et al., 2012). The internal standard used to calculate the lipid and fatty acid content in the samples was heptadecanoic acid. Results are expressed in μg of the sum of UFA per mg of FW, and are presented as means ± SD of three biological replicates.

## Results and Discussion

### Olive *SAD* Genes Expression and Unsaturated Fatty Acids Composition Are Regulated by Temperature

To investigate how temperature regulates UFA synthesis in olive mesocarp, olive branches from Picual and Arbequina cultivars holding olive fruits at 28 WAF, which correspond to turning stage, were incubated at low (15°C) and high (35°C) temperature with a 12 h light/12 h dark cycle, for 24 h. Lipid and *SAD* genes expression analysis were carried out using olive mesocarp at different times after incubation at the aforementioned conditions.

**FIGURE 1:**
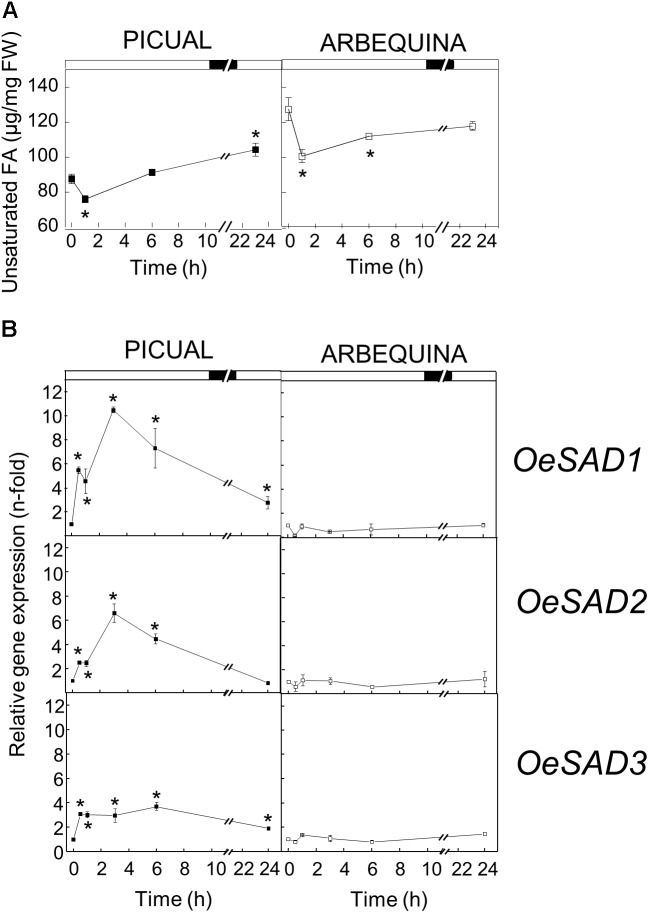
Effect of low temperature on the unsaturated fatty acids content **(A)** and the relative expression levels of olive *SAD1*, *SAD2*, and *SAD3* genes **(B)** in the mesocarp tissue from cultivars Picual and Arbequina. Branches with about 100 olive fruit (28 WAF) were incubated using standard conditions except that the temperature was 15°C. At the indicated times, fatty acid composition was analyzed by gas chromatography, and relative expressions levels were determined by qRT-PCR using the expression level of the corresponding gene at zero time as calibrator. Data are presented as means ± SD of three biological replicates. ^∗^Indicates significantly different to time 0 h (*p* < 0.05) by two-way ANOVA with a Bonferroni post-test.

The effect of low temperature (15°C) on the UFA content and *SAD* transcript levels was different in the two cultivars studied. Low temperatures reduced the UFA content at the beginning of incubation in both cultivars, and then recovered practically to the initial levels at 6 h of incubation. These levels were maintained in Arbequina mesocarp until the end of the experiment. However, in Picual mesocarp the UFA levels increased during the 24 h period, reaching about 20% more than at the beginning of the treatment (Figure 1A). These results correlated well with the expression levels of *SAD* genes in mesocarp tissue exposed to the cold treatment. In this way, while Arbequina *SAD* transcripts remained practically constant throughout the treatment, in Picual mesocarp the expression levels of the three *SAD* genes showed a transient and significant increase during the first 3 h of incubation, with *SAD1* undergoing a 10-fold increase in gene expression compared to *SAD2* and *SAD*3 with 6- and 3-fold increase, respectively (Figure 1B). The effect of incubation at 15°C observed in Picual mesocarp is the general response to low temperature changes for desaturases, since there are numerous reports describing that low temperature causes an increase in the UFA content (Los and Murata, 1998). With only two exceptions in lima bean (Zhang et al., 2011) and avocado (García-Rojas et al., 2012), an induction of *SAD* genes by cold stress has been reported in different plant species. For instance, Wang et al. (2013) described that in *Ginkgo biloba* leaves, *SAD* mRNA levels increased transiently, reaching a maximum at 6 h after incubation at 4 or 15°C, and then decreased after 24 h, analogous to what we observed in the Picual olive mesocarp. Similarly, a transient induction of *SAD* gene was observed in tea leaves incubated at 4 or −5°C for 24 h (Ding et al., 2016). In a like manner, an increase in *SAD* transcript in response to low temperature has been described in avocado fruit (Madi et al., 2003), potato leaves (Vega et al., 2004; De Palma et al., 2008), rape hypocotyl (Tasseva et al., 2004), and soybean seed (Byfield and Upchurch, 2007), which was also accompanied by an increment in the UFA content. The role of *SAD* gene in plant cold stress has also been demonstrated in transplastomic tobacco plants expressing a wild potato *SAD* gene, that exhibited increased UFA content and improved cold tolerance (Craig et al., 2008). Furthermore, the rice cold inducible transcription factor Osmyb4, which is involved in the cold stress response, has been shown to transactivate the wild potato *SAD* gene promoter (Vannini et al., 2004).

To gain further insight about the cold stress response observed in olive mesocarp from Picual cultivar, we decided to analyze the UFA content in the different lipids classes from this tissue incubated at 15°C for 24 h, to investigate whether the detected increase in *SAD* genes expression leads to an increase in the UFA content in membrane lipids. We observed that the UFA content in triacylglycerol decreased throughout the incubation at 15°C, while UFA levels increased significantly in the membrane phospholipids (Table 1). In addition, we observed an increase in all of the UFA, being oleic acid the one that increases the most (Supplementary Table S2). These data suggested that low temperature incubation induced a mobilization of UFA from storage lipids into microsomal membrane lipids.

**Table 1:** Effect of low temperature on the unsaturated fatty acids content of lipid classes from Picual mesocarp tissue.

Lipid class	Unsaturated fatty acid content (μg/mg FW)			
	0	1	6	24
DAG	0.467 ± 0.064	0.481 ± 0.011	0.534 ± 0.065	0.562 ± 0.025
TAG	82.403 ± 1.165	68.520 ± 0.131^∗^	66.309 ± 3.813^∗^	56.945 ± 8.220^∗^
PI	0.010 ± 0.002	0.012 ± 0.002	0.014 ± 0.001	0. 024 ± 0.001
PS	0.004 ± 0.001	0.005 ± 0.001	0.011 ± 0.000	0. 011 ± 0.003
PC	0.049 ± 0.002	0.045 ± 0.004	0.070 ± 0.003	0. 068 ± 0.004
PE	0.005 ± 0.002	0.018 ± 0.000	0.021 ± 0.005	0. 021 ± 0.005
PA	0.027 ± 0.002	0.044 ± 0.002	0.052 ± 0.003	0. 046 ± 0.002
MGDG	0.045 ± 0.001	0.053 ± 0.002	0.037 ± 0.004	0.046 ± 0.003
DGDG	0.025 ± 0.002	0.049 ± 0.001	0.030 ± 0.002	0.040 ± 0.005

*DAG, diacylglycerol; TAG, triacylglycerol; PI, phosphatidylinositol; PS, phosphatidylserine; PC, phosphatidylcholine; PE, phosphatidylethanolamine; PA, phosphatidate; MGDG, monogalactosyldiacylglycerol; DGDG, digalactosyldiacylglycerol. Data are mean ± SD from three biological replicates. ^∗^Indicates significantly different to time 0 h (p < 0.05) by two-way ANOVA with a Bonferroni post-test.*

We have previously reported an induction of *FAD2* genes in mesocarp tissue incubated at 15°C for 24 h, although non-significant differences were detected in the linoleic acid content of microsomal and plastidial membranes (Hernández et al., 2011). In that study, we suggested that the timescale could be too short to observe effects, although the existence of post-transcriptional regulatory mechanism could not be discarded. In this work (Supplementary Table S2), we noticed that the increase in oleic acid in membrane phospholipids took place from the beginning of the cold treatment (1 h after incubation), whereas a very slight increase of linoleic acid was detected in phosphatidic acid, phosphatidylserine, phosphatidylcholine, and phosphatidylethanolamine at longer times (6–24 h after incubation), suggesting that low temperature induced the synthesis of oleic acid in the short–term, while the increase of linoleic acid occurs at longer incubations periods. These results indicate that the observed changes in the transcript levels of *SAD* genes caused by low temperature are accompanied by the adjustment of UFA content of microsomal membrane lipids and modulation of membrane fluidity in olive mesocarp cv. Picual.

The fact that low temperatures did not increase either *SAD* expression levels or the UFA levels in Arbequina mesocarp could be related with a lower cold tolerance of this cultivar with respect to Picual. In this sense, Vega et al. (2004) reported that the expression of *SAD* gene increased during cold acclimation only in *Solanum commersonii*, a species capable of cold acclimation, and not in the cultivated non-cold acclimating *Solanum tuberosum* species, although the latter had a greater amount of constitutive *SAD* gene expression, indicating that the changes in transcript accumulation observed in *S. commersonii* may be related to its capacity to cold acclimate. However, these data contrast slightly with those found by De Palma et al. (2008), which provided evidence that the freezing tolerant *S. commersonii* plants have a higher constitutive transcript levels of *SAD* gene. Nevertheless, these results altogether suggest that *SAD* transcript accumulation plays a key role in cold tolerance. This assumption was further confirmed by Li et al. (2015), who reported that *SAD* overexpression caused an increase in membrane linoleic acid content, which improved the cold acclimation capacity of transgenic potato plants.

**FIGURE 2:**
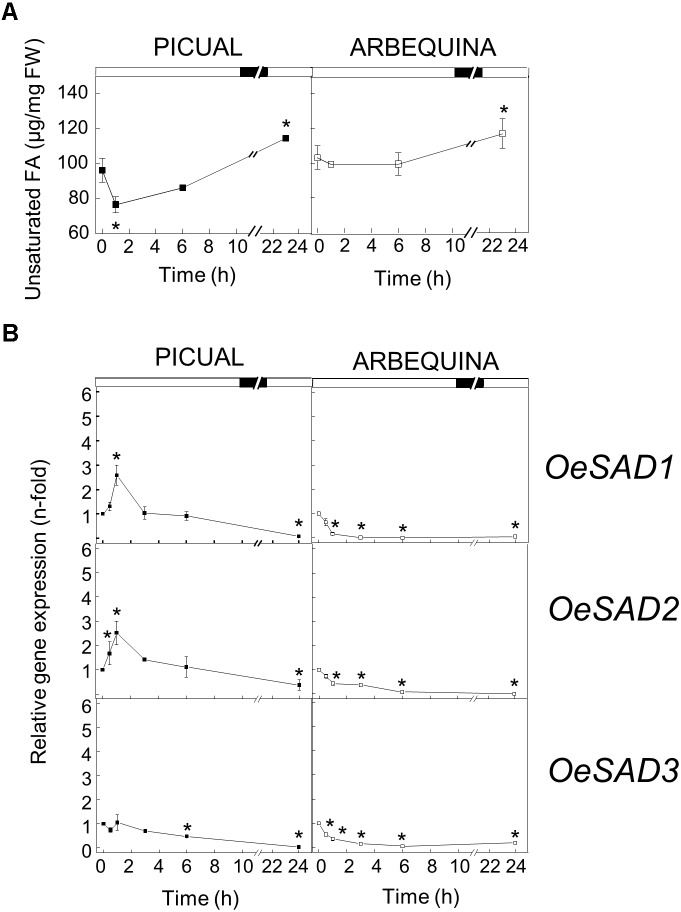
Effect of high temperature on the unsaturated fatty acids content **(A)** and the relative expression levels of olive *SAD1*, *SAD2*, and *SAD3* genes **(B)** in the mesocarp tissue from cultivars Picual and Arbequina. Branches with about 100 olive fruit (28 WAF) were incubated using standard conditions except that the temperature was 35°C. At the indicated times, fatty acid composition was analyzed by gas chromatography, and relative expressions levels were determined by qRT-PCR using the expression level of the corresponding gene at zero time as calibrator. Data are presented as means ± SD of three biological replicates. ^∗^Indicates significantly different to time 0 h (*p* < 0.05) by two-way ANOVA with a Bonferroni post-test.

With respect to high temperature, when we incubated the olive branches with turning olive fruits at 35°C for 24 h, we observed a decreased in the UFA content 1 h after the incubation, to subsequently recover to the initial values throughout the incubation period in both cultivars, reaching even higher values than the initial ones after 24 h of incubation (Figure 2A). The changes observed in UFA content are mainly detected in TAG for both cultivars (Supplementary Tables S3, S4). These modifications in UFA levels did not correlate well with the *SAD* genes expression pattern detected in mesocarp tissue exposed to high temperature. We observed in Figure 2B that the expression levels of the three *SAD* genes decreased during the incubation at 35°C, although *SAD* genes expression patterns were different in both cultivars. Specifically, in cv. Picual, *SAD1* and *SAD2* transcripts slightly increased at 0.5 and 1 h of incubation, the three genes expression levels were similar to initial values at 3 h, and then decreased considerably after 24 h of incubation. However, in cv. Arbequina the three *SAD* transcripts decreased from the beginning of the treatment. The downregulation of *SAD* genes has been previously reported by Wang et al. (2013) in leaves of ginkgo grown at 35 or 45°C, and by Lu et al. (2013) in *Pinellia ternata* leaves incubated at 35°C for 24 h. In addition, when soybean plants were incubated under warm conditions, the *SAD-A* and *SAD-B* genes expression levels decreased in the seeds, but with negligible effect on the seed stearate content (Byfield and Upchurch, 2007). The lack of correlation between the effect of high temperature on *SAD* genes expression levels and the UFA content could be explained by several factors. In particular, the high temperature regulation of oleic acid synthesis may be mediated by post-transcriptional mechanism. Several cases of temperature-related post-transcriptional mechanisms have been reported for the oleate desaturases. In sunflower seeds, it has been described that changes in temperature bring about shifts in the very low endogenous oxygen concentration, which affect FAD2 activity reversibly, without having an effect on gene transcription (Rolletschek et al., 2007). Besides, Tang et al. (2005) identified two domains that appear to be important in mediating the temperature-dependent instability of the soybean FAD2-1A isoform when expressed in yeast. However, to date, no mechanism of post-transcriptional regulation by temperature has been described for SAD enzymes.

### Regulation of Olive *SAD* Transcript Levels and Unsaturated Fatty Acids Content by Darkness

**FIGURE 3:**
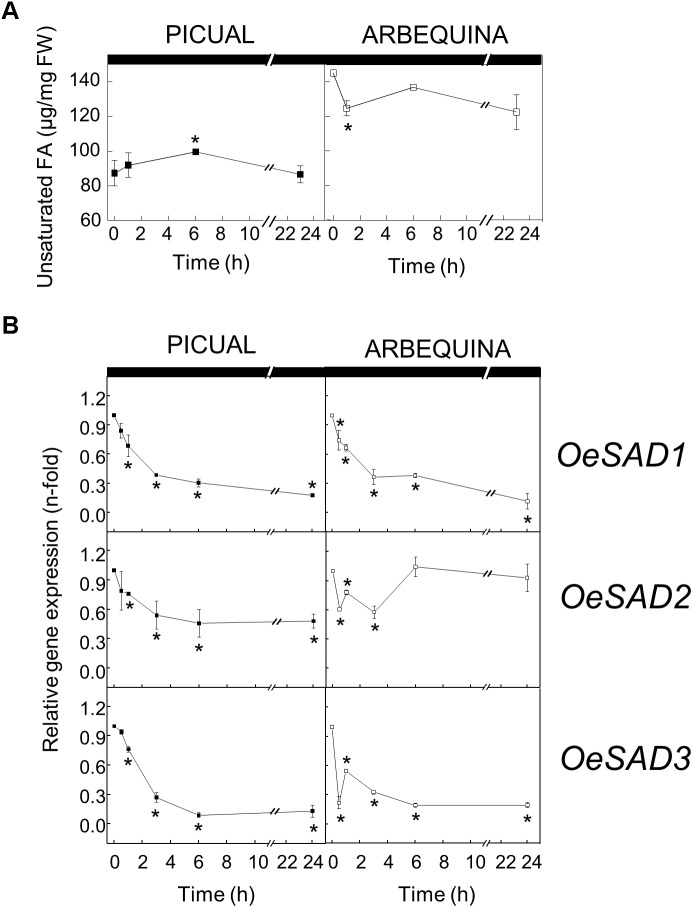
Effect of darkness on the unsaturated fatty acids content **(A)** and the relative expression levels of olive *SAD1*, *SAD2*, and *SAD3* genes **(B)** in the mesocarp tissue from cultivars Picual and Arbequina. Branches with about 100 olive fruit (28 WAF) were incubated at 25°C under darkness conditions. At the indicated times, fatty acid composition was analyzed by gas chromatography, and relative expressions levels were determined by qRT-PCR using the expression level of the corresponding gene at zero time as calibrator. Data are presented as means ± SD of three biological replicates. ^∗^Indicates significantly different to time 0 h (*p* < 0.05) by two-way ANOVA with a Bonferroni post-test.

To test whether darkness alters the UFA content and *SAD* transcript levels in olive mesocarp, olive branches from Picual and Arbequina cultivars holding olive fruits at 28 WAF (turning stage) were incubated at 25°C in the darkness, for 24 h. Although we did not observe any significant difference in the total UFA content during the incubation period, a decrease in the three *SAD* genes expression levels was detected in both cultivars (Figure 3). *SAD1* and *SAD3* transcripts levels decreased considerably in both cultivars from the beginning of the treatment, so that after 24 h of incubation reached a reduction of 90-fold in comparison to the initial levels. However, the decrease in *SAD2* expression levels was about 50-fold after 3 h of incubation, maintaining these levels in Picual cultivar and recovering the initial values in the case of Arbequina (Figure 3B).

Several evidences indicate that light may play a regulatory role for plant desaturases, although data are scarce in this area. A light-dependent transcriptional regulation of oleate desaturase genes has been described in olive mesocarp (Hernández et al., 2011), similarly to that reported by Kargiotidou et al. (2008) in cotton cotyledons, where *FAD2* expression was reduced after a 24 h incubation in the dark. In the same way, *FAD7* and *FAD8* are also transcriptionally regulated by light in different plants (Nishiuchi et al., 1995; Horiguchi et al., 1996; Collados et al., 2006). In general, it appears that increasing light conditions are associated with an enhancement in desaturases genes expression, with the converse also holding true in darkness conditions. Although this is the first time that the effect of darkness on *SAD* genes expression levels is reported in plants, the regulatory role of light on Δ9 desaturase genes has been studied before in other photosynthetic organism. Kis et al. (1998) described that in the cyanobacteria *Synechocystis* PCC 6803 desaturases genes were strongly induced by light, except for the Δ9 desaturase, that was not significantly affected. On the other hand, Ma et al. (2018) observed that the expression levels of *SAD* gene from the green microalgae *Haematococcus pluvialis* were significantly upregulated by light, which correlated well with an increase in oleic acid. These results are in agreement with the decrease in *SAD* genes expression levels observed in olive mesocarp in darkness conditions, although we did not observe a decrease in the total UFA content.

**FIGURE 4:**
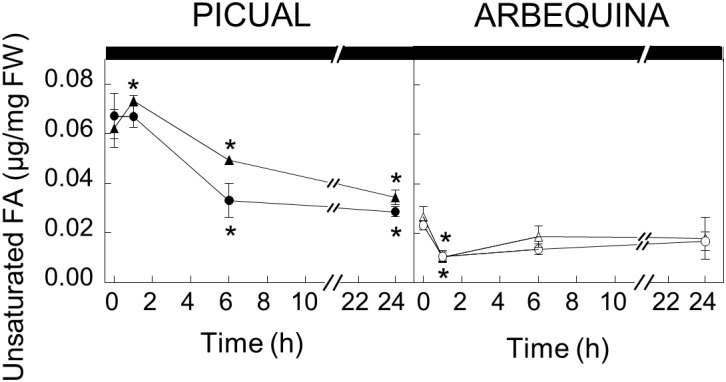
Effect of darkness on the galactolipids unsaturated fatty acid content in the mesocarp tissue from cultivars Picual and Arbequina. Branches with about 100 olive fruit (28 WAF) were incubated at 25°C under darkness conditions. At the indicated times, fatty acid composition of galactolipids were analyzed by gas chromatography a Triangles, monogalactosyldiacylglycerol; circles, digalactosyldiacylglycerol. Data are presented as means ± SD of three biological replicates. ^∗^Indicates significantly different to time 0 h (*p* < 0.05) by two-way ANOVA with a Bonferroni post-test.

To further investigate the effect of darkness in oleic acid synthesis, we decided to analyze the UFA content of the different lipid classes in olive mesocarp subjected to darkness conditions, in order to elucidate whether the decrease in the expression levels of *SAD* genes affects the UFA content of a specific lipid. Interestingly, we only observed a decrease in the UFA content of the galactolipids, MGDG and DGDG, in both cultivars (Figure 4), although with different behavior. While in Picual mesocarp, the UFA content decreased considerably during the 24 h of incubation under darkness conditions, in Arbequina mesocarp, the UFA levels were reduced after 1 h of incubation, and then recovered, almost reaching the levels detected at the beginning of the treatment. This effect of the dark on the pattern of UFA content in galactolipids correlated quite well with that of *SAD2* expression levels in both cultivars and not with *SAD1* and *SAD3*, which suggests, that the reduction of *SAD2* transcript due to the dark incubation could be the responsible for the decrease in UFA in galactolipids. In fact, we noticed that the decrease detected in the UFA content of MGDG and DGDG during the dark incubation of olive mesocarp is mainly due to a reduction in oleic acid content (Table 2). Therefore, we can conclude that the down-regulation of *SAD2* gene during the dark incubation is the responsible for the reduction in the UFA content in the main chloroplast lipids, MGDG and DGDG. Furthermore, this light-dependent regulation detected in olive mesocarp is cultivar-dependent, since the restoration of *SAD2* gene expression levels and UFA content in galactolipids after 24 h of incubation observed in Arbequina cultivar, was not detected in Picual mesocarp. The fact that the effect of darkness was noticeable on chloroplast-localized lipids is not unexpected. Gemmrich (1982) observed in *Ricinus communis* cultures, that the light-induced changes in lipid composition were associated with thylakoid formation. Strong light induces ultrastructural changes in chloroplasts, so that the area of thylakoid system on chloroplast sections increases, there is an accumulation of chloroplast-localized lipids, like MGDG, and the unsaturation index of fatty acids is elevated, being the relative content of linolenic acid the one that increases the most (Kislyuk et al., 2013).

**Table 2:** Effect of darkness on the fatty acid composition of galactolipids from Picual and Arbequina mesocarp tissue.

Cultivar	Lipid class	Time (h)	Fatty acid composition (μg/mg FW)					
			16:0	16:1	18:0	18:1	18:2	18:3
Picual	MGDG	0	0.015 ± 0.002	0.002 ± 0.001	0.006 ± 0.000	0.054 ± 0.005	0.004 ± 0.000	0.000 ± 0.000
		1	0.016 ± 0.002	0.002 ± 0.000	0.006 ± 0.001	0.065 ± 0.002^∗^	0.005 ± 0.000	0.000 ± 0.000
		6	0.014 ± 0.002^∗^	0.001 ± 0.000	0.007 ± 0.001	0.044 ± 0.001^∗^	0.003 ± 0.000	0.000 ± 0.000
		24	0.010 ± 0.002	0.001 ± 0.000	0.008 ± 0.000	0.030 ± 0.002^∗^	0.002 ± 0.000	0.000 ± 0.000
	DGDG	0	0.027 ± 0.001	0.001 ± 0.000	0.010 ± 0.004	0.060 ± 0.010	0.003 ± 0.000	0.001 ± 0.000
		1	0.021 ± 0.003	0.001 ± 0.000	0.007 ± 0.000	0.060 ± 0.004	0.003 ± 0.000	0.002 ± 0.000
		6	0.014 ± 0.001^∗^	0.001 ± 0.000	0.006 ± 0.000	0.029 ± 0.007^∗^	0.002 ± 0.000	0.001 ± 0.000
		24	0.016 ± 0.001^∗^	0.001 ± 0.000	0.008 ± 0.001	0.025 ± 0.003^∗^	0.001 ± 0.000	0.001 ± 0.000
Arbequina	MGDG	0	0.007 ± 0.002	0.000 ± 0.000	0.018 ± 0.002	0.023 ± 0.003	0.003 ± 0.001	0.000 ± 0.000
		1	0.008 ± 0.000	0.000 ± 0.000	0.012 ± 0.003	0.008 ± 0.002^∗^	0.002 ± 0.001	0.000 ± 0.000
		6	0.008 ± 0.000	0.000 ± 0.000	0.014 ± 0.001	0.014 ± 0.003^∗^	0.004 ± 0.001	0.000 ± 0.000
		24	0.010 ± 0.002	0.000 ± 0.000	0.012 ± 0.001	0.014 ± 0.006^∗^	0.003 ± 0.002	0.000 ± 0.000
	DGDG	0	0.008 ± 0.001	0.000 ± 0.000	0.016 ± 0.002	0.022 ± 0.002	0.001 ± 0.000	0.000 ± 0.000
		1	0.008 ± 0.000	0.000 ± 0.000	0.011 ± 0.001	0.009 ± 0.002^∗^	0.001 ± 0.000	0.000 ± 0.000
		6	0.009 ± 0.002	0.000 ± 0.000	0.013 ± 0.001	0.012 ± 0.002^∗^	0.002 ± 0.000	0.000 ± 0.000
		24	0.009 ± 0.001	0.000 ± 0.000	0.012 ± 0.001	0.014 ± 0.003^∗^	0.002 ± 0.001	0.000 ± 0.000

*Data are presented as means ± SD of three biological replicates. MGDG, monogalactosyldiacylglycerol; DGDG, digalactosyldiacylglycerol. 16:0, palmitic acid;16:1, palmitoleic acid; 18:0, stearic acid; 18:1 oleic acid; 18:2, linoleic acid; 18:3, linolenic acid. ^∗^Indicates significantly different to time 0 h (p < 0.05) by two-way ANOVA with a Bonferroni post-test.*

### Differential Transcriptional Regulation of Olive *SAD* Genes in Response to Wounding

**FIGURE 5:**
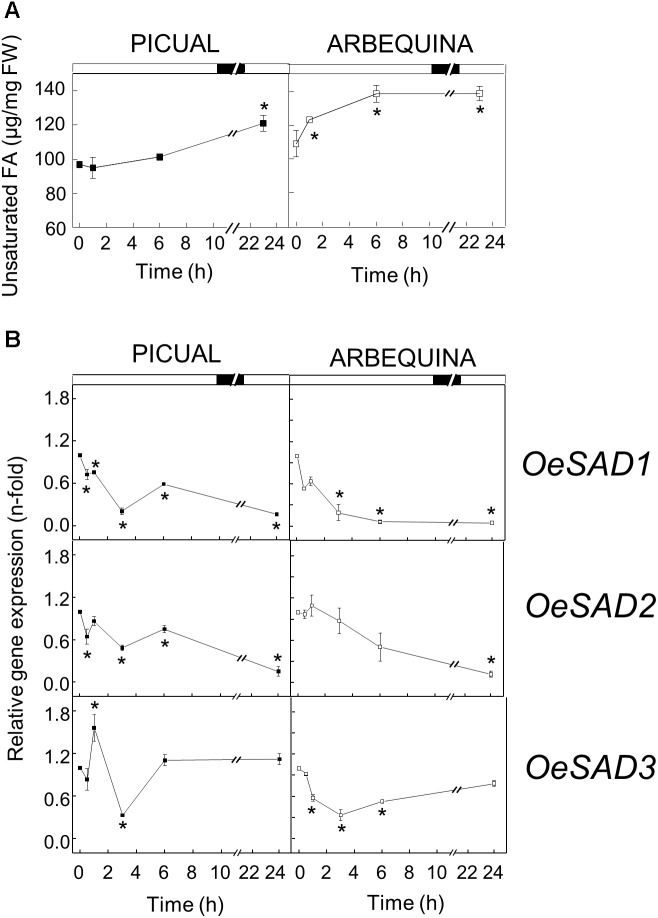
Effect of wounding on the unsaturated fatty acids content **(A)** and the relative expression levels of olive *SAD1*, *SAD2* and *SAD3* genes **(B)** in the mesocarp tissue from cultivars Picual and Arbequina. Branches with about 100 olive fruit (28 WAF) were incubated using standard conditions except that the olive fruit were mechanically damaged at zero time. At the indicated times, fatty acid composition was analyzed by gas chromatography, and relative expressions levels were determined by qRT-PCR using the expression level of the corresponding gene at zero time as calibrator. Data are presented as means ± SD of three biological replicates. ^∗^Indicates significantly different to time 0 h (*p* < 0.05) by two-way ANOVA with a Bonferroni post-test.

The involvement of a Δ9 stearate desaturase in defense mechanisms was firstly demonstrated by Xing and Chin (2000), who reported that the expression of a yeast Δ9 desaturase in eggplant enhanced its resistance to *Verticillium dahliae*. To investigate possible changes in the UFA composition and *SAD* genes expression of olive mesocarp in response to wounding, olive branches from Picual and Arbequina cultivars holding olive fruits at 28 WAF (turning stage) were incubated using standard conditions except that olive fruit were mechanically damaged at zero time. A slight increase in the UFA content was observed when lipid analysis was performed at different times of incubation after wounding, although with some cultivar differences. While in cv. Picual the increase in UFA content after wounding was slow and progressive, in Arbequina mesocarp the UFA content increases rapidly after 1 h of incubation, continues rising until 6 h and then, the levels were maintained until the end of the incubation (Figure 5A).

In contrast, except in the case of Picual *SAD3* gene, olive *SAD* genes transcript levels decreased after wounding in both cultivars (Figure 5B). It has been demonstrated that a mutation in the Arabidopsis gene *ssi2/fab2*, which encodes a SAD, resulted in the reduction of oleic acid levels, causing the constitutive defense response in the mutant plant (Kachroo and Kachroo, 2009; Xia et al., 2009). Analogous results were observed in *OsSSI2*-knockdown plants in rice (Jiang et al., 2009), and in *GmSAD*-silenced soybean (Kachroo et al., 2008). In addition, overexpression of the *TaSSI2* in *ssi2* Arabidopsis mutant plants resulted in restoration of oleic acid and, thereby, rescued other *ssi2*-associated phenotypes (Song et al., 2013). Further studies revealed that the reduced oleic acid levels triggered the transcriptional up-regulation of pathogenesis-related genes, the genes governing synthesis of salicylic acid, and nitric oxide responsive nuclear genes, thus activating disease resistance (Venugopal et al., 2009; Mandal et al., 2012). In this sense, we also detected in olive mesocarp a discrete reduction of 1.24% in Picual and 2.88% in Arbequina in oleic acid levels after wounding (Table 3). Since olive *SAD2* has been reported to be the main gene contributing to the oleic acid content in olive mesocarp (Parvini et al., 2016), the down-regulation of olive *SAD1* and *SAD2* genes observed in response to wounding could be responsible for the decrease in the oleic acid proportion and, consequently, trigger the defense response.

**Table 3:** Effect of wounding on the fatty acid composition of Picual and Arbequina mesocarp tissue.

Cultivar	Time(h)	Fatty acid composition (%)						
		16:0	16:1	16:2	18:0	18:1	18:2	18:3
Picual	0	13.41 ± 0.04	1.27 ± 0.04	0.10 ± 0.01	2.24 ± 0.00	80.19 ± 0.03	2.17 ± 0.01	0.62 ± 0.01
	1	13.71 ± 0.06 ^∗^	1.62 ± 0.03^∗^	0.09 ± 0.00	2.17 ± 0.10	79.47 ± 0.04 ^∗^	2.36 ± 0.05 ^∗^	0.58 ± 0.01
	6	13.86 ± 0.03^∗^	1.65 ± 0.00^∗^	0.10 ± 0.01	2.37 ± 0.01 ^∗^	78.87 ± 0.04 ^∗^	2.54 ± 0.01 ^∗^	0.61 ± 0.00
	24	13.57 ± 0.06 ^∗^	1.51 ± 0.01^∗^	0.11 ± 0.00	2.19 ± 0.01	78.95 ± 0.09 ^∗^	3.13 ± 0.01 ^∗^	0.55 ± 0.00
Arbequina	0	17.48 ± 0.40	2.02 ± 0.06	0.26 ± 0.02	1.83 ± 0.04	66.14 ± 0.83	11.70 ± 0.36	0.57 ± 0.03
	1	18.53 ± 0.10 ^∗^	2.33 ± 0.02	0.33 ± 0.01	1.75 ± 0.01	62.62 ± 0.32 ^∗^	13.81 ± 0.10 ^∗^	0.63 ± 0.11
	6	18.15 ± 0.06 ^∗^	2.38 ± 0.01	0.27 ± 0.02	1.81 ± 0.01	65.01 ± 0.04 ^∗^	11.78 ± 0.03	0.59 ± 0.11
	24	18.24 ± 0.10 ^∗^	2.19 ± 0.03	0.33 ± 0.02	1.78 ± 0.00	63.26 ± 0.19 ^∗^	13.62 ± 0.13 ^∗^	0.59 ± 0.07

*Data are presented as means ± SD of three biological replicates. 16:0, palmitic acid; 16:1, palmitoleic acid; 16:2, palmitolinoleic acid; 18:0, stearic acid; 18:1, oleic acid; 18:2, linoleic acid; 18:3, α-linolenic acid. ^∗^Indicates significantly different to time 0 h (p < 0.05) by two-way ANOVA with a Bonferroni post-test.*

Unlike *SAD1* and *SAD2* genes, *SAD3* increased its transcript levels transiently after 1 h in cv. Picual, and returned to initial values at 24 h of treatment (Figure 5B). Remarkably, *SAD3* gene has been previously shown to be induced in olive leaves infected by *Spilocaea oleagina* (Benítez et al., 2005), and *Verticillium dahliae* and *Fusarium* spp. (Trabelsi et al., 2017). Similar results to olive *SAD3* gene induction were observed in yellow lupine, avocado and tea. Zaborowska et al. (2002) reported an increase in *SAD* gene transcripts in yellow lupine nodules from 12 days after infection with *Bradyrhizobium* sp. (*Lupinus*). In the same way, *SAD* expression levels increased in response to wounding in avocado fruit (Madi et al., 2003) and tea leaves (Ding et al., 2016). Interestingly, we observed that the reduction in the oleic acid levels was accompanied by an increase in palmitolinoleic and linoleic acids at 24 h after wounding in both cultivars (Table 3). We have reported before this increase in dienoic fatty acids induced by wounding, showing that *FAD2* genes are involved in the wounding response of olive fruit mesocarp, and causing an increase in the content of palmitolinoleic and linoleic acids in microsomal lipids (Hernández et al., 2011). In the same study, we also suggested that the synthesis of palmitolinoleic acid is a consequence of the simultaneous induction of *SAD* and *FAD2* genes in olive fruit mesocarp in response to wounding. Since, in this work, we have observed a specific induction of the *SAD3* gene (Figure 5B), it is tempting to speculate that the SAD3 isoform is the one involved in this response mechanism. These dienoic fatty acids probably serve as precursors of a different set of oxylipins involved in plant defense, generated by the lipoxygenase pathway (Weber, 2002).

## Conclusion

In the present study, we have demonstrated that low temperature transcriptionally regulates *SAD* genes from olive mesocarp in a cultivar-dependent manner, leading to a modification of the UFA content in Picual microsomal membrane lipids, in order to maintain membrane fluidity in the mesocarp tissue. On the contrary, in the case of high temperature *SAD* genes expression levels did not correlate well with the UFA content in olive mesocarp. Our results have also shown that the decrease of *SAD* gene transcripts caused by darkness in olive mesocarp was accompanied by a reduction in the UFA content of chloroplast lipids. In addition, the differential transcriptional regulation of *SAD* genes after wounding seems to have a crucial role in the olive defense response, not only by reducing oleic acid levels, which triggers the transcriptional up-regulation of defense related genes, but also by promoting the increase of dienoic fatty acids, that serve as precursors of oxylipins. Taken together, the data presented in this work point out that the different environmental stresses can modify the content of oleic acid and its polyunsaturated derivatives in the olive mesocarp through the transcriptional regulation of *SAD* genes, affecting olive oil quality.

## Data Availability

All datasets generated for this study are included in the manuscript and/or the Supplementary Files.

## Author Contributions

MH managed and performed the stress experiments, carried out RNA isolation and cDNA synthesis, and drafted the manuscript. MS performed the qRT-PCR and lipid analysis. MA revised the study and the manuscript. JM-R conceived and designed the study and contributed to manuscript revision. All authors discussed, commented and approved the final version of the manuscript.

## Conflict of Interest Statement

The authors declare that the research was conducted in the absence of any commercial or financial relationships that could be construed as a potential conflict of interest.

## Abbreviations

DGDG: digalactosyldiacylglycerol
FAD2: microsomal oleate desaturase
FAD3: microsomal linoleate desaturase
FAD6: plastidial oleate desaturase
FAD7/8: plastidial linoleate desaturase
MGDG: monogalactosyldiacylglycerol
SAD: stearoyl-acyl carrier protein desaturase
UFA: unsaturated fatty acids
WAF: weeks after flowering.
