# Supplementary material for: Transcriptional Regulation of Stearoyl-Acyl Carrier Protein Desaturase Genes in Response to Abiotic Stresses Leads to Changes in the Unsaturated Fatty Acids Composition of Olive Mesocarp
Source: Front Plant Sci. 2019 Mar 5;10:251. doi: 10.3389/fpls.2019.00251 (PMC6411816; doi:10.3389/fpls.2019.00251)
Supplement: Supplementary file 1 [file Presentation_1.PPTX]

## Slide 1
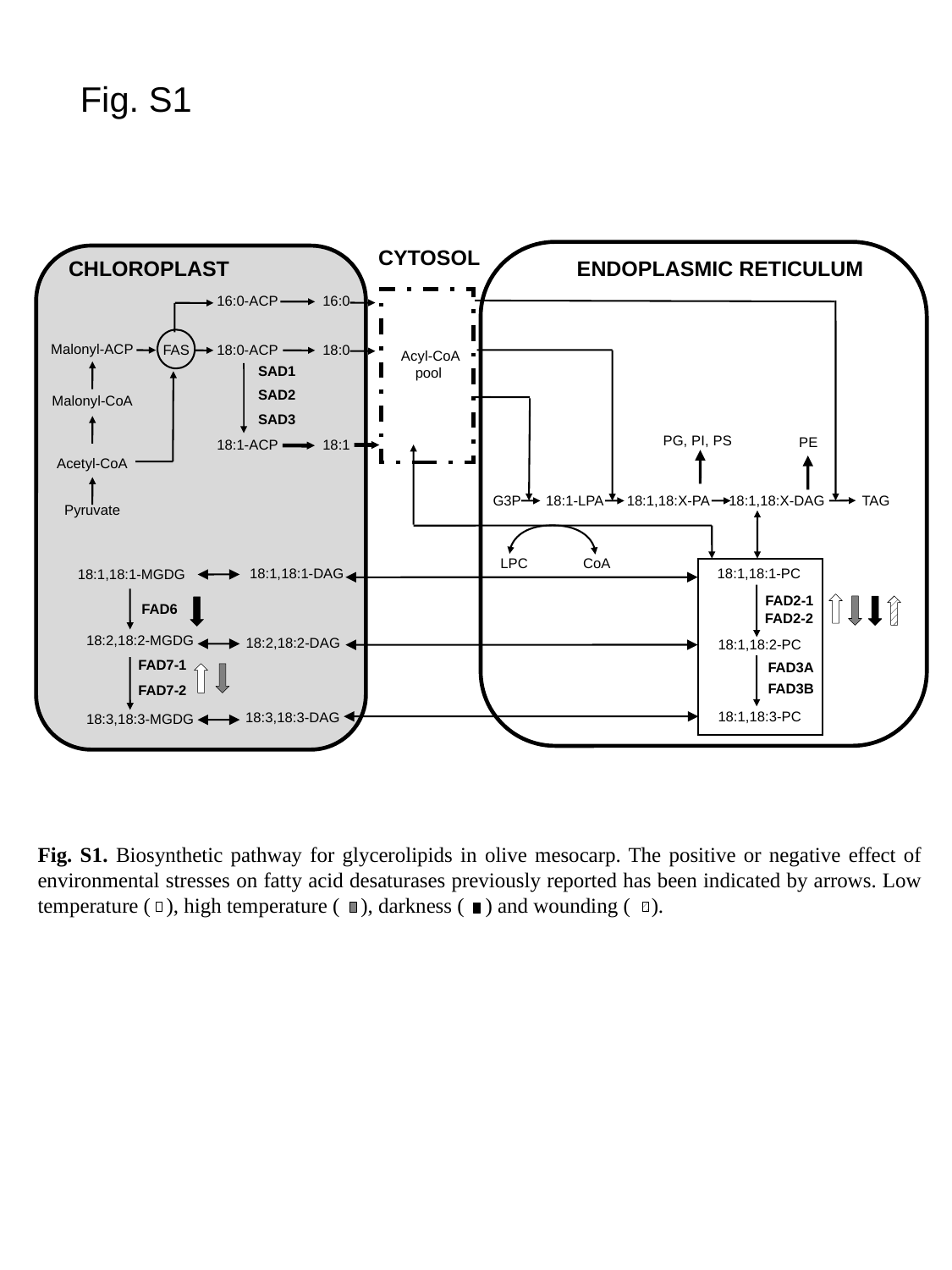

Fig. S1
CYTOSOL
CHLOROPLAST
ENDOPLASMIC RETICULUM
16:0-ACP
16:0
 Acyl-CoA
pool
Malonyl-ACP
18:0-ACP
18:0
FAS
SAD1
SAD2
SAD3
Malonyl-CoA
PG, PI, PS
PE
18:1-ACP
18:1
Acetyl-CoA
G3P
G3P
18:1-LPA
18:1,18:X-PA
18:1,18:X-DAG
TAG
Pyruvate
LPC
CoA
18:1,18:1-DAG
18:1,18:1-PC
18:1,18:1-MGDG
FAD2-1
FAD2-1
FAD6
FAD2-2
18:2,18:2-MGDG
18:2,18:2-DAG
18:1,18:2-PC
FAD7-1
FAD3A
FAD3A
FAD3B
FAD7-2
18:1,18:3-PC
18:3,18:3-DAG
18:3,18:3-MGDG
Fig. S1. Biosynthetic pathway for glycerolipids in olive mesocarp. The positive or negative effect of environmental stresses on fatty acid desaturases previously reported has been indicated by arrows. Low temperature ( ), high temperature ( ), darkness ( ) and wounding ( ).

## Slide 2
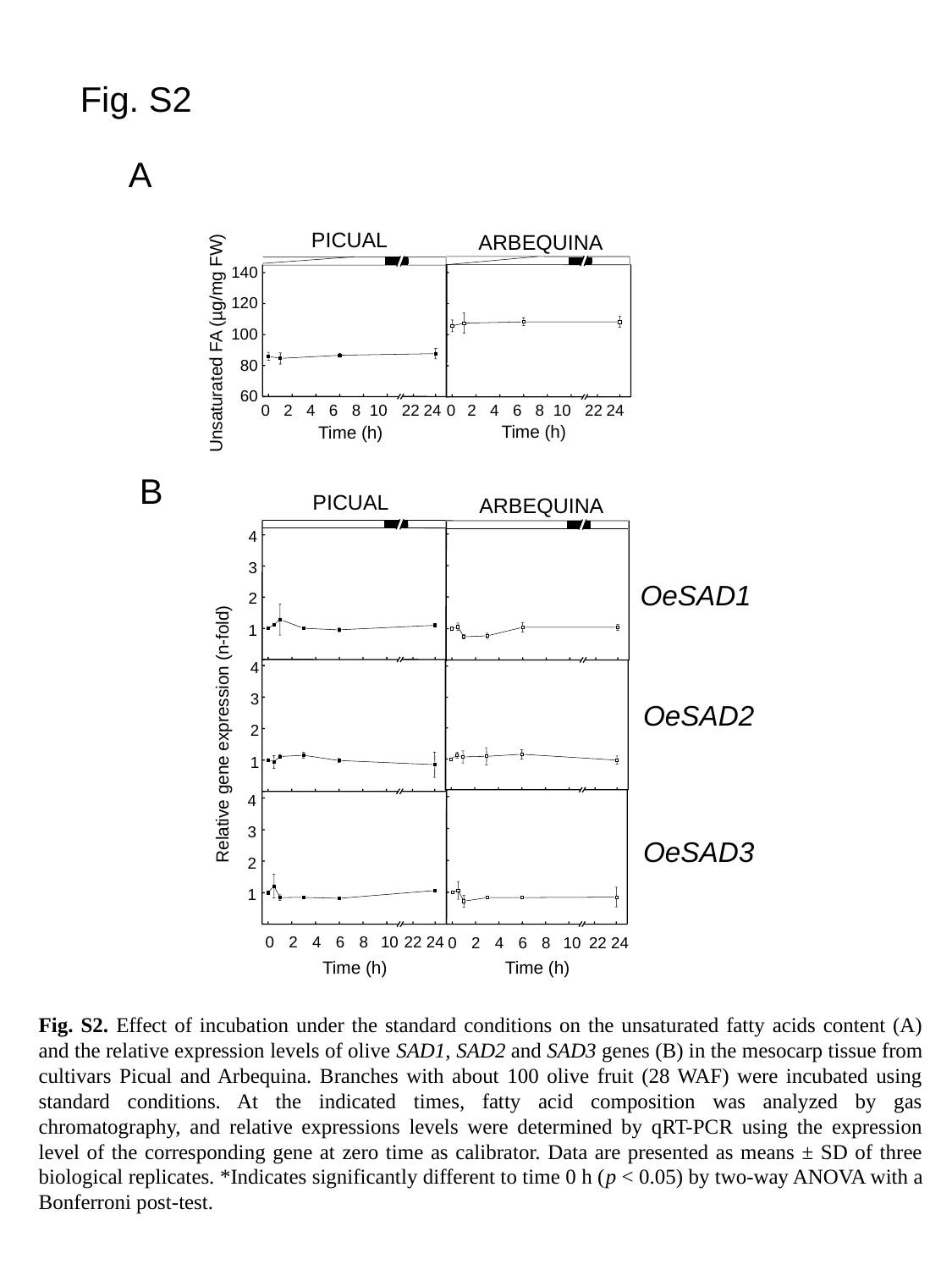

Fig. S2
A
PICUAL
ARBEQUINA
140
120
100
Unsaturated FA (µg/mg FW)
80
60
0
2
4
6
8
10
22
24
0
2
4
6
8
10
22
24
Time (h)
Time (h)
B
PICUAL
ARBEQUINA
4
3
OeSAD1
2
1
4
3
OeSAD2
2
Relative gene expression (n-fold)
1
4
3
OeSAD3
2
1
0
2
4
6
8
10
22
24
0
2
4
6
8
10
22
24
Time (h)
Time (h)
Fig. S2. Effect of incubation under the standard conditions on the unsaturated fatty acids content (A) and the relative expression levels of olive SAD1, SAD2 and SAD3 genes (B) in the mesocarp tissue from cultivars Picual and Arbequina. Branches with about 100 olive fruit (28 WAF) were incubated using standard conditions. At the indicated times, fatty acid composition was analyzed by gas chromatography, and relative expressions levels were determined by qRT-PCR using the expression level of the corresponding gene at zero time as calibrator. Data are presented as means ± SD of three biological replicates. *Indicates significantly different to time 0 h (p < 0.05) by two-way ANOVA with a Bonferroni post-test.
