# Supplementary material for: Transcriptional Regulation of Stearoyl-Acyl Carrier Protein Desaturase Genes in Response to Abiotic Stresses Leads to Changes in the Unsaturated Fatty Acids Composition of Olive Mesocarp
Source: Front Plant Sci. 2019 Mar 5;10:251. doi: 10.3389/fpls.2019.00251 (PMC6411816; doi:10.3389/fpls.2019.00251)
Supplement: Supplementary file 2 [file Presentation_2.pptx]

## Slide 1
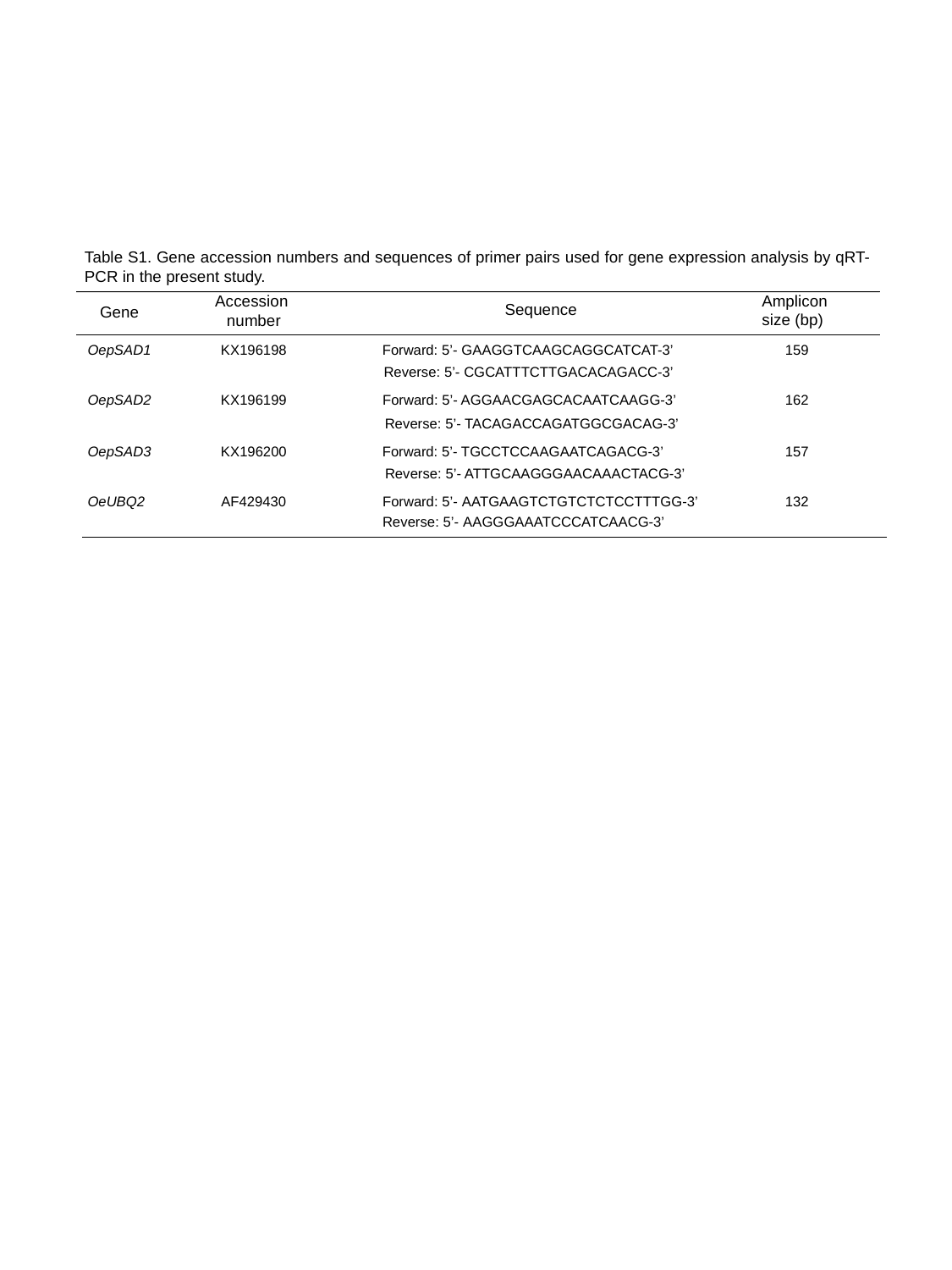

Table S1. Gene accession numbers and sequences of primer pairs used for gene expression analysis by qRT-PCR in the present study.
Amplicon
size (bp)
Accession
number
Sequence
Gene
OepSAD1
KX196198
Forward: 5’- GAAGGTCAAGCAGGCATCAT-3’
159
Reverse: 5’- CGCATTTCTTGACACAGACC-3’
OepSAD2
KX196199
Forward: 5’- AGGAACGAGCACAATCAAGG-3’
162
Reverse: 5’- TACAGACCAGATGGCGACAG-3’
OepSAD3
KX196200
Forward: 5’- TGCCTCCAAGAATCAGACG-3’
157
Reverse: 5’- ATTGCAAGGGAACAAACTACG-3’
OeUBQ2
AF429430
Forward: 5’- AATGAAGTCTGTCTCTCCTTTGG-3’
132
Reverse: 5’- AAGGGAAATCCCATCAACG-3’

## Slide 2
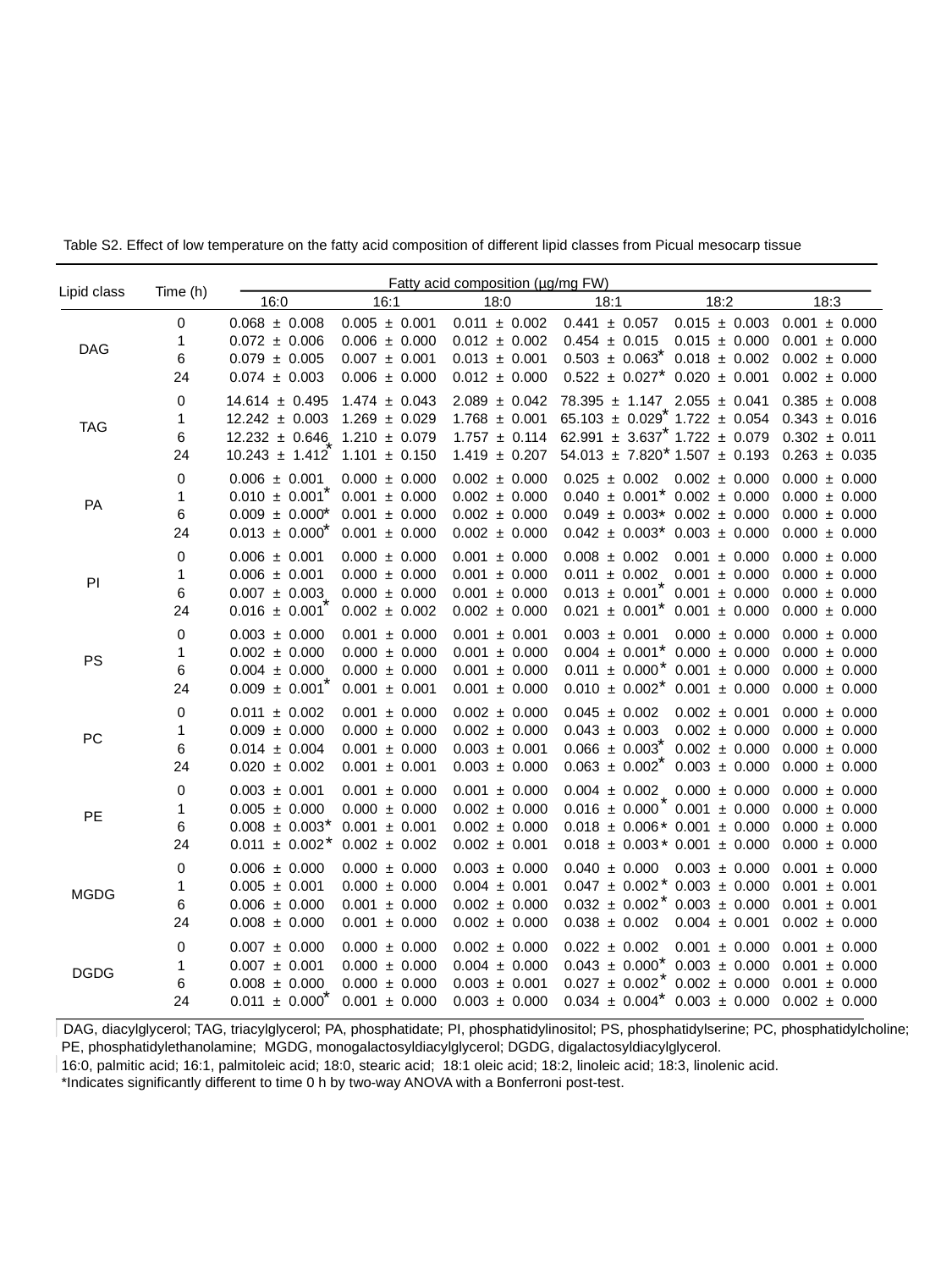

Table S2. Effect of low temperature on the fatty acid composition of different lipid classes from Picual mesocarp tissue
Fatty acid composition (µg/mg FW)
Lipid class
Time (h)
16:0
16:1
18:0
18:1
18:2
18:3
0
0.068 ± 0.008
0.005 ± 0.001
0.011 ± 0.002
0.441 ± 0.057
0.015 ± 0.003
0.001 ± 0.000
1
0.072 ± 0.006
0.006 ± 0.000
0.012 ± 0.002
0.454 ± 0.015
0.015 ± 0.000
0.001 ± 0.000
*
DAG
6
0.079 ± 0.005
0.007 ± 0.001
0.013 ± 0.001
0.503 ± 0.063
0.018 ± 0.002
0.002 ± 0.000
*
24
0.074 ± 0.003
0.006 ± 0.000
0.012 ± 0.000
0.522 ± 0.027
0.020 ± 0.001
0.002 ± 0.000
0
14.614 ± 0.495
1.474 ± 0.043
2.089 ± 0.042
78.395 ± 1.147
2.055 ± 0.041
0.385 ± 0.008
*
1
12.242 ± 0.003
1.269 ± 0.029
1.768 ± 0.001
65.103 ± 0.029
1.722 ± 0.054
0.343 ± 0.016
*
TAG
6
12.232 ± 0.646
1.210 ± 0.079
1.757 ± 0.114
62.991 ± 3.637
1.722 ± 0.079
0.302 ± 0.011
*
*
24
10.243 ± 1.412
1.101 ± 0.150
1.419 ± 0.207
54.013 ± 7.820
1.507 ± 0.193
0.263 ± 0.035
0
0.006 ± 0.001
0.000 ± 0.000
0.002 ± 0.000
0.025 ± 0.002
0.002 ± 0.000
0.000 ± 0.000
*
*
1
0.010 ± 0.001
0.001 ± 0.000
0.002 ± 0.000
0.040 ± 0.001
0.002 ± 0.000
0.000 ± 0.000
PA
*
*
6
0.009 ± 0.000
0.001 ± 0.000
0.002 ± 0.000
0.049 ± 0.003
0.002 ± 0.000
0.000 ± 0.000
*
*
24
0.013 ± 0.000
0.001 ± 0.000
0.002 ± 0.000
0.042 ± 0.003
0.003 ± 0.000
0.000 ± 0.000
0
0.006 ± 0.001
0.000 ± 0.000
0.001 ± 0.000
0.008 ± 0.002
0.001 ± 0.000
0.000 ± 0.000
1
0.006 ± 0.001
0.000 ± 0.000
0.001 ± 0.000
0.011 ± 0.002
0.001 ± 0.000
0.000 ± 0.000
*
PI
6
0.007 ± 0.003
0.000 ± 0.000
0.001 ± 0.000
0.013 ± 0.001
0.001 ± 0.000
0.000 ± 0.000
*
*
24
0.016 ± 0.001
0.002 ± 0.002
0.002 ± 0.000
0.021 ± 0.001
0.001 ± 0.000
0.000 ± 0.000
0
0.003 ± 0.000
0.001 ± 0.000
0.001 ± 0.001
0.003 ± 0.001
0.000 ± 0.000
0.000 ± 0.000
*
1
0.002 ± 0.000
0.000 ± 0.000
0.001 ± 0.000
0.004 ± 0.001
0.000 ± 0.000
0.000 ± 0.000
*
PS
6
0.004 ± 0.000
0.000 ± 0.000
0.001 ± 0.000
0.011 ± 0.000
0.001 ± 0.000
0.000 ± 0.000
*
*
24
0.009 ± 0.001
0.001 ± 0.001
0.001 ± 0.000
0.010 ± 0.002
0.001 ± 0.000
0.000 ± 0.000
0
0.011 ± 0.002
0.001 ± 0.000
0.002 ± 0.000
0.045 ± 0.002
0.002 ± 0.001
0.000 ± 0.000
1
0.009 ± 0.000
0.000 ± 0.000
0.002 ± 0.000
0.043 ± 0.003
0.002 ± 0.000
0.000 ± 0.000
*
PC
6
0.014 ± 0.004
0.001 ± 0.000
0.003 ± 0.001
0.066 ± 0.003
0.002 ± 0.000
0.000 ± 0.000
*
24
0.020 ± 0.002
0.001 ± 0.001
0.003 ± 0.000
0.063 ± 0.002
0.003 ± 0.000
0.000 ± 0.000
0
0.003 ± 0.001
0.001 ± 0.000
0.001 ± 0.000
0.004 ± 0.002
0.000 ± 0.000
0.000 ± 0.000
*
1
0.005 ± 0.000
0.000 ± 0.000
0.002 ± 0.000
0.016 ± 0.000
0.001 ± 0.000
0.000 ± 0.000
PE
*
*
6
0.008 ± 0.003
0.001 ± 0.001
0.002 ± 0.000
0.018 ± 0.006
0.001 ± 0.000
0.000 ± 0.000
*
*
24
0.011 ± 0.002
0.002 ± 0.002
0.002 ± 0.001
0.018 ± 0.003
0.001 ± 0.000
0.000 ± 0.000
0
0.006 ± 0.000
0.000 ± 0.000
0.003 ± 0.000
0.040 ± 0.000
0.003 ± 0.000
0.001 ± 0.000
*
1
0.005 ± 0.001
0.000 ± 0.000
0.004 ± 0.001
0.047 ± 0.002
0.003 ± 0.000
0.001 ± 0.001
*
MGDG
6
0.006 ± 0.000
0.001 ± 0.000
0.002 ± 0.000
0.032 ± 0.002
0.003 ± 0.000
0.001 ± 0.001
24
0.008 ± 0.000
0.001 ± 0.000
0.002 ± 0.000
0.038 ± 0.002
0.004 ± 0.001
0.002 ± 0.000
0
0.007 ± 0.000
0.000 ± 0.000
0.002 ± 0.000
0.022 ± 0.002
0.001 ± 0.000
0.001 ± 0.000
*
1
0.007 ± 0.001
0.000 ± 0.000
0.004 ± 0.000
0.043 ± 0.000
0.003 ± 0.000
0.001 ± 0.000
*
DGDG
6
0.008 ± 0.000
0.000 ± 0.000
0.003 ± 0.001
0.027 ± 0.002
0.002 ± 0.000
0.001 ± 0.000
*
*
24
0.011 ± 0.000
0.001 ± 0.000
0.003 ± 0.000
0.034 ± 0.004
0.003 ± 0.000
0.002 ± 0.000
DAG, diacylglycerol; TAG, triacylglycerol; PA, phosphatidate; PI, phosphatidylinositol; PS, phosphatidylserine; PC, phosphatidylcholine;
PE, phosphatidylethanolamine; MGDG, monogalactosyldiacylglycerol; DGDG, digalactosyldiacylglycerol.
16:0, palmitic acid; 16:1, palmitoleic acid; 18:0, stearic acid; 18:1 oleic acid; 18:2, linoleic acid; 18:3, linolenic acid.
*Indicates significantly different to time 0 h by two-way ANOVA with a Bonferroni post-test.

## Slide 3
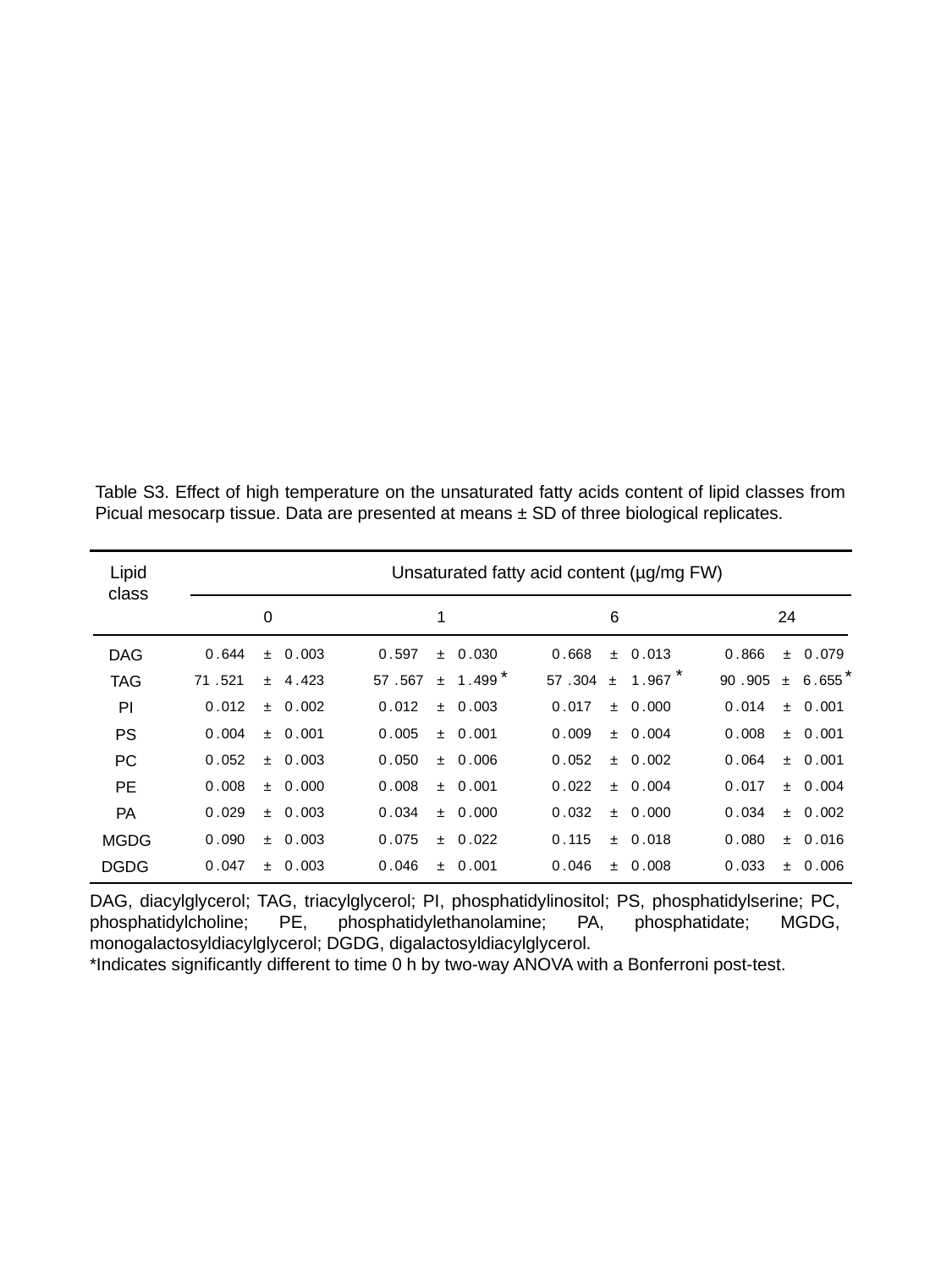

Table S3. Effect of high temperature on the unsaturated fatty acids content of lipid classes from Picual mesocarp tissue. Data are presented at means ± SD of three biological replicates.
Lipid
class
Unsaturated fatty acid content (µg/mg FW)
0
1
6
24
DAG
0
.
644
±
0
.
003
0
.
597
±
0
.
030
0
.
668
±
0
.
013
0
.
866
±
0
.
079
*
*
*
TAG
71
.
521
±
4
.
423
57
.
567
±
1
.
499
57
.
304
±
1
.
967
90
.
905
±
6
.
655
PI
0
.
012
±
0
.
002
0
.
012
±
0
.
003
0
.
017
±
0
.
000
0
.
014
±
0
.
001
PS
0
.
004
±
0
.
001
0
.
005
±
0
.
001
0
.
009
±
0
.
004
0
.
008
±
0
.
001
PC
0
.
052
±
0
.
003
0
.
050
±
0
.
006
0
.
052
±
0
.
002
0
.
064
±
0
.
001
PE
0
.
008
±
0
.
000
0
.
008
±
0
.
001
0
.
022
±
0
.
004
0
.
017
±
0
.
004
PA
0
.
029
±
0
.
003
0
.
034
±
0
.
000
0
.
032
±
0
.
000
0
.
034
±
0
.
002
MGDG
0
.
090
±
0
.
003
0
.
075
±
0
.
022
0
.
115
±
0
.
018
0
.
080
±
0
.
016
DGDG
0
.
047
±
0
.
003
0
.
046
±
0
.
001
0
.
046
±
0
.
008
0
.
033
±
0
.
006
DAG, diacylglycerol; TAG, triacylglycerol; PI, phosphatidylinositol; PS, phosphatidylserine; PC, phosphatidylcholine; PE, phosphatidylethanolamine; PA, phosphatidate; MGDG, monogalactosyldiacylglycerol; DGDG, digalactosyldiacylglycerol.
*Indicates significantly different to time 0 h by two-way ANOVA with a Bonferroni post-test.

## Slide 4
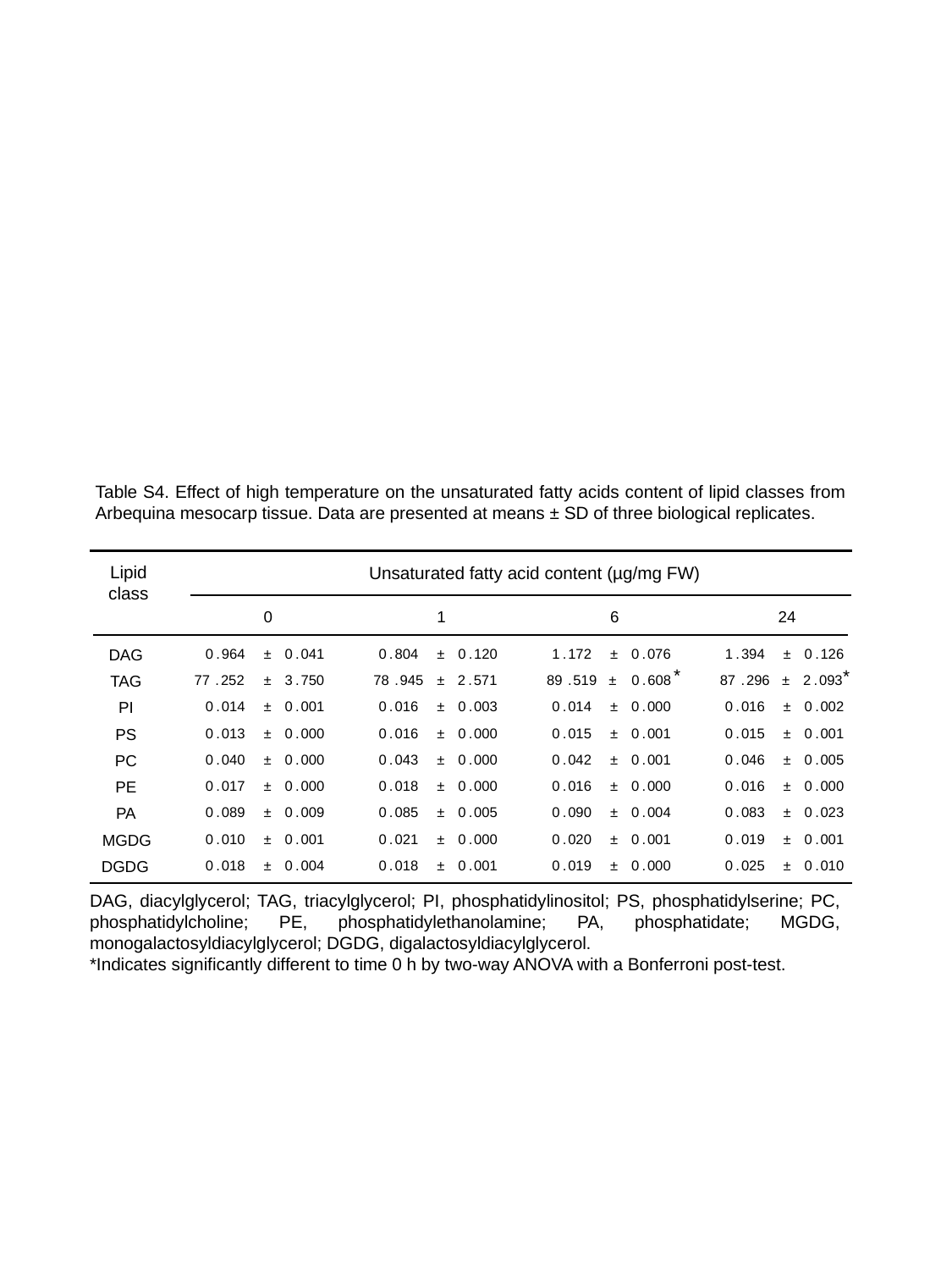

Table S4. Effect of high temperature on the unsaturated fatty acids content of lipid classes from Arbequina mesocarp tissue. Data are presented at means ± SD of three biological replicates.
Lipid
class
Unsaturated fatty acid content (µg/mg FW)
0
1
6
24
DAG
0
.
964
±
0
.
041
0
.
804
±
0
.
120
1
.
172
±
0
.
076
1
.
394
±
0
.
126
*
*
TAG
77
.
252
±
3
.
750
78
.
945
±
2
.
571
89
.
519
±
0
.
608
87
.
296
±
2
.
093
PI
0
.
014
±
0
.
001
0
.
016
±
0
.
003
0
.
014
±
0
.
000
0
.
016
±
0
.
002
PS
0
.
013
±
0
.
000
0
.
016
±
0
.
000
0
.
015
±
0
.
001
0
.
015
±
0
.
001
PC
0
.
040
±
0
.
000
0
.
043
±
0
.
000
0
.
042
±
0
.
001
0
.
046
±
0
.
005
PE
0
.
017
±
0
.
000
0
.
018
±
0
.
000
0
.
016
±
0
.
000
0
.
016
±
0
.
000
PA
0
.
089
±
0
.
009
0
.
085
±
0
.
005
0
.
090
±
0
.
004
0
.
083
±
0
.
023
MGDG
0
.
010
±
0
.
001
0
.
021
±
0
.
000
0
.
020
±
0
.
001
0
.
019
±
0
.
001
DGDG
0
.
018
±
0
.
004
0
.
018
±
0
.
001
0
.
019
±
0
.
000
0
.
025
±
0
.
010
DAG, diacylglycerol; TAG, triacylglycerol; PI, phosphatidylinositol; PS, phosphatidylserine; PC, phosphatidylcholine; PE, phosphatidylethanolamine; PA, phosphatidate; MGDG, monogalactosyldiacylglycerol; DGDG, digalactosyldiacylglycerol.
*Indicates significantly different to time 0 h by two-way ANOVA with a Bonferroni post-test.
